# Glycemic Outcomes Persist for up to 2 Years in Very Young Children with the Omnipod^®^ 5 Automated Insulin Delivery System

**DOI:** 10.1089/dia.2023.0506

**Published:** 2024-06-30

**Authors:** Daniel J. DeSalvo, Bruce W. Bode, Gregory P. Forlenza, Lori M. Laffel, Bruce A. Buckingham, Amy B. Criego, Melissa Schoelwer, Sarah A. MacLeish, Jennifer L. Sherr, David W. Hansen, Trang T. Ly

**Affiliations:** ^1^ Department of Pediatrics, Baylor College of Medicine, Houston, Texas, USA.; ^2^ Atlanta Diabetes Associates, Atlanta, Georgia, USA.; ^3^ Department of Pediatrics, Barbara Davis Center for Diabetes, University of Colorado Anschutz Medical Campus, Aurora, Colorado, USA.; ^4^ Joslin Diabetes Center, Harvard Medical School, Boston, Massachusetts, USA.; ^5^ Division of Pediatric Endocrinology, Department of Pediatrics, Stanford University, Stanford, California, USA.; ^6^ International Diabetes Center-HealthPartners Institute, Park Nicollet Pediatric Endocrinology, Minneapolis, Minnesota, USA.; ^7^ Center for Diabetes Technology, University of Virginia, Charlottesville, Virginia, USA.; ^8^ Department of Pediatrics, University Hospitals Cleveland Medical Center, Rainbow Babies and Children's Hospital, Cleveland, Ohio, USA.; ^9^ Department of Pediatrics, Yale School of Medicine, New Haven, Connecticut, USA.; ^10^ Department of Pediatrics, SUNY Upstate Medical University, Syracuse, New York, USA.; ^11^ Clinical Affairs Department, Insulet Corporation, Acton, Massachusetts, USA.

**Keywords:** Type 1 diabetes, Artificial pancreas, Insulin pumps, Clinical trials, Closed-loop systems, Pediatric diabetes

## Abstract

**Background:**

To evaluate the long-term safety and effectiveness of the Omnipod^®^ 5 Automated Insulin Delivery (AID) System in very young children with type 1 diabetes with up to 2 years of use.

**Methods:**

Following a 13-week single-arm, multicenter, pivotal trial that took place after 14 days of standard therapy data collection, participating children (2–5.9 years of age at study enrollment) were provided the option to continue use of the AID system in an extension phase. HbA1c was measured every 3 months, up to 15 months of total use, and continuous glucose monitor metrics were collected through the completion of the extension study (for up to 2 years).

**Results:**

Participants (*N* = 80) completed 18.2 [17.4, 23.4] (median [interquartile range]) total months of AID, inclusive of the 3-month pivotal trial. During the pivotal trial, HbA1c decreased from 7.4% ± 1.0% (57 ± 10.9 mmol/mol) to 6.9% ± 0.7% (52 ± 7.7 mmol/mol, *P* < 0.0001) and was maintained at 7.0% ± 0.7% (53 ± 7.7 mmol/mol) after 15 months total use (*P* < 0.0001 from baseline). Time in target range (70–180 mg/dL) increased from 57.2% ± 15.3% during standard therapy to 68.1% ± 9.0% during the pivotal trial (*P* < 0.0001) and was maintained at 67.2% ± 9.3% during the extension phase (*P* < 0.0001 from standard therapy). Participants spent a median 97.1% of time in Automated Mode during the extension phase, with one episode of severe hypoglycemia and one episode of diabetic ketoacidosis.

**Conclusion:**

This evaluation of the Omnipod 5 AID System indicates that long-term use can safely maintain improvements in glycemic outcomes with up to 2 years of use in very young children with type 1 diabetes.

**Clinical Trials Registration Number:** NCT04476472

## Introduction

Young children ages 2–5.9 years with type 1 diabetes are a population who struggle with achieving optimal glycemic outcomes, which remains crucial for neurocognitive development and avoidance of long-term complications.^1,2^ With a limited cognitive skillset at this age, these children require full-time care from family and/or caregivers. This can be overwhelming and burdensome not only for the child, but also for those that play a large role in their life, with the greatest concerns occurring at night when severe hypoglycemia could go undetected.^3,4^ Due to the unique circumstances of this population, automated insulin delivery (AID) systems that aim to safely improve glycemia are crucial to relieving burden and reducing parents' concerns. Studies on the impact of AID systems in very young children are limited and are particularly sparse when it comes to studying their long-term impact.^5–10^

The Omnipod^®^ 5 AID System is comprised of two components: a wearable, tubeless insulin-filled pump with a built-in algorithm (Pod) and the Omnipod 5 App as a controller and is interoperable with a compatible continuous glucose monitor (CGM), currently the Dexcom G6. The effectiveness and safety of the Omnipod 5 AID System has been previously assessed in a sample of children who were 2–5.9 years of age at study enrollment in a 3-month single-arm, multicenter pivotal trial, which demonstrated an improvement of 10.9% ± 9.6%, or more than 2½ h daily, time in range (TIR) 70–180 mg/dL when using Omnipod 5 compared with standard therapy.^11^ Reductions in HbA1c, hypoglycemia, and hyperglycemia were also reported. Furthermore, there were no episodes of diabetic ketoacidosis or severe hypoglycemia during the 3-month pivotal trial.^11^ While these short-term outcomes are encouraging, there is a need to assess whether improved outcomes with AID systems in these young children are durable over time, especially given the rapid growth and lifestyle changes experienced at this age.

To address this gap, we evaluated the long-term safety and effectiveness of the Omnipod 5 AID System in very young children with type 1 diabetes who were 2–5.9 years of age at enrollment for up to 2 years of at-home use.

## Materials and Methods

This extension study is a continuation of a 3-month outpatient pivotal trial of the Omnipod 5 AID System (Insulet Corporation, Acton, MA) in very young children, details of which have been published previously.^11^ The pivotal trial included 80 participants (children 2 to 5.9 years of age at enrollment) with type 1 diabetes (any duration) with an HbA1c <10% (86 mmol/mol) at screening (complete eligibility criteria in Supplementary Table S1). The participants completed a 14-day standard therapy phase using their usual insulin therapy (multiple daily injections [MDI] or insulin pump) with glycemic outcomes measured by CGM, which was blinded for those not already using the Dexcom G6 as part of their usual therapy, followed by a 13-week AID phase using the Omnipod 5 AID System. Pivotal trial participants were then provided the option to continue use of the Omnipod 5 AID System as part of this extension study.

### Study conduct and oversight

A central Institutional Review Board and local Review Boards approved the protocol. Oversight was provided by an independent Data and Safety Monitoring Board. Informed consent was provided by each participant's parent/guardian. The United States Food and Drug Administration approved an investigational device exemption for use in the pivotal trial and the subsequent extension phase. The trial was registered at clinicaltrials.gov (NCT04476472).

### Study design and participants

Every participant (100%, 80/80) who took part in the pivotal trial completed the study and chose to enter the optional extension phase with continued use of the Omnipod 5 AID System. This resulted in a total of 80 children (2–5.9 years of age at pivotal trial enrollment) beginning use of AID between September 8, 2020 and October 22, 2020, and beginning the extension phase between December 7, 2020 and January 21, 2021.

This was an open-ended study design with a variable duration lasting until the system could be obtained commercially, which varied based on participant age. The Omnipod 5 AID System FDA clearance was received for ages 6 years and older on January 27, 2022, at which point participants who were 6 years of age and older at the time of clearance were transitioned out of the study and onto the commercial system as desired. The remaining participants less than 6 years old remained in the extension phase until they turned 6 years old or until the Omnipod 5 AID System FDA clearance was received for ages 2 and older on August 19, 2022. The last participant visit was on October 12, 2022. This resulted in an extension phase duration of median [interquartile range, IQR] 15.2 [14.5, 20.3] months for a total use of 18.2 [17.6, 23.3] months, with a maximum of 24.7 months (inclusive of the 3-month pivotal trial phase).

For the extension phase, follow-up visits occurred once every 30 days for the first six visits following completion of the pivotal study and then were spaced further apart to every 45 days for all remaining visits (Supplementary Table S2). Laboratory assessments of HbA1c were conducted every 3 months (with the first measurement having been measured at baseline before system use) for up to 15 months of total Omnipod 5 AID System use, after which no further HbA1c measurements were taken. Glucose sensor and device use outcomes were measured through the entirety of the extension phase for each participant (up to 2 years of total use). Each follow-up visit assessed medication use, adverse events, and device deficiencies or complaints, reported by the child's parent/guardian. Device data were automatically and continuously uploaded and subsequently reviewed during each scheduled visit to assess safety.

### Investigational device

This investigational device includes two main components: a tubeless on-body insulin pump with an embedded AID algorithm (Pod) and a mobile application on a locked-down Android phone. This investigational device is interoperable with a CGM (Dexcom G6; Dexcom, San Diego). The built-in AID algorithm utilizes current and predicted glucose values to deliver insulin micro-boluses every 5 min to reach the user-set target glucose value (user-configurable from 110 to 150 mg/dL in 10 mg/dL increments, with up to eight segments per day). Meal and correction boluses can be delivered by the user through the Bolus Calculator within the Omnipod 5 App. Additional details on the device features were previously published.^12^

### Outcomes

Primary effectiveness endpoints were HbA1c during the extension phase as compared with baseline HbA1c and percentage TIR during the extension phase as compared with the standard therapy phase. Primary safety endpoints were incidence rates of both severe hypoglycemia and diabetic ketoacidosis. The secondary objective was to evaluate endpoints, including glucose metrics from CGM (e.g., mean glucose, standard deviation [SD], and coefficient of variation; percent of time <54 mg/dL, <70 mg/dL, >180 mg/dL, ≥250 mg/dL, and ≥300 mg/dL), clinical metrics (e.g., body mass index [BMI], total daily insulin, total daily basal and bolus insulin), and system use metrics (e.g., percent of time in Automated Mode and number of device deficiencies).

### Statistical methods

Analyses were performed using a modified intention-to-treat dataset of participants who entered the extension phase. Glycemic endpoints were tested using paired *t*-tests or Wilcoxon signed-rank tests (Wilcoxon signed-rank tests used for groups with <10 participants or if Shapiro–Wilk tests of normality were significant [*P* < 0.05]). All *P*-values were considered significant at a two-sided significance level of 5%. Correction of *P*-values for multiple comparisons was not conducted. Continuous variables were summarized using count, mean, median, SD, and range; categorical variables were summarized by frequencies and percentages. No imputations were completed for missing data. SAS version 9.4 was used for conducting this analysis.

## Results

### Participants

From December 7, 2020, to January 5, 2021, all participants (80 children, baseline characteristics provided in Table 1) elected to continue into the optional extension phase following completion of the 3-month pivotal trial. Ninety-nine percent (79/80) of participants remained in the extension phase until the system became commercially available, based on their current age. Thirty-one percent of participants used the system for at least 23 months with 25% of participants using the system for at least 24 months of total use. Baseline characteristics of the 20 participants using the system for at least 24 months (Table 1) were similar to those of the overall study population; however, these participants were slightly younger (mean ± SD age of 3.6 ± 0.7 years compared with 4.7 ± 1.0 years overall). The duration of the extension study varied as participants were able to transition out of the study and onto the commercial system once it was cleared for their age group, and due to participants starting the pivotal phase earlier or later in the enrollment period. All but one participant (78/79) completing the extension study planned to transition to the commercially available system.

**Table 1.** Characteristics at Baseline for Those Electing to Participate in the Extension Phase

Characteristic	All participants	Participants with ≥2 years of total system use
*N*	80	20
Age, years	4.7 ± 1.0 (2.0, 6.0^a^)	3.6 ± 0.7 (2.5, 4.5)
Duration of diabetes, years	2.3 ± 1.1 (0.1, 4.6)	2.0 ± 0.8 (0.5, 3.9)
Body mass index^b^	16.7 ± 1.5 (14.0, 21.7)	16.9 ± 1.9 (14.4, 21.7)
Female sex, *n* (%)	34 (42.5)	8 (40.0)
Race/ethnicity, *n* (%)^c^		
White	67 (83.8)	18 (90.0)
Hispanic or Latino	5 (6.3)	2 (10.0)
Not Hispanic or Latino	62 (77.5)	16 (80.0)
Black or African American	4 (5.0)	2 (10.0)
Asian	3 (3.8)	—
Other	1 (1.3)	—
Hispanic or Latino	1 (1.3)	—
Not Hispanic or Latino	—	—
Two or more races	5 (6.3)	—
Hispanic or Latino	1 (1.3)	—
Not Hispanic or Latino	4 (5.0)	—
HbA1c, %^d^	7.4 ± 1.0 (5.4, 10.2)	7.3 ± 0.9 (6.1, 9.5)
HbA1c, mmol/mol^d^	57 ± 10.9 (36, 88)	56 ± 9.8 (43, 80)
Daily insulin dose, U/kg^e^	0.69 ± 0.18 (0.30, 1.33)	0.69 ± 0.17 (0.39, 0.97)
Previous^f^ or current continuous glucose monitor use, *n* (%)	78 (97.5)	19 (95.0)
Previous^f^ or current pump use, *n* (%)	68 (85.0)	18 (90.0)
Using multiple daily injections as standard therapy method, *n* (%)	12 (15.0)	2 (10.0)

Plus–minus values are means ± SD. Unless otherwise indicated, remaining values are range (minimum, maximum).

^a^ Age was determined at the date of informed consent for the pivotal study. Values are rounded to the nearest 10th.

^b^ Body mass index is the weight in kilograms divided by the square of the height in meters.

^c^ Race and ethnicity were reported by the participants and are displayed exactly as reported. As shown, several participants chose more than one racial category. Ethnicity delineation is shown for racial categories where at least one person identified as Hispanic or Latino.

^d^ Participant eligibility for the study was determined using a point-of-care HbA1c measurement performed at screening, which in some cases differed from the laboratory assessment displayed here and used for analysis.

^e^ Baseline total daily insulin dose was determined from data collected during the standard therapy phase.

^f^ Previous use is defined as having used the device for any duration in the past.

SD, standard deviation.

### Glycemic outcomes

**FIG. 1.**
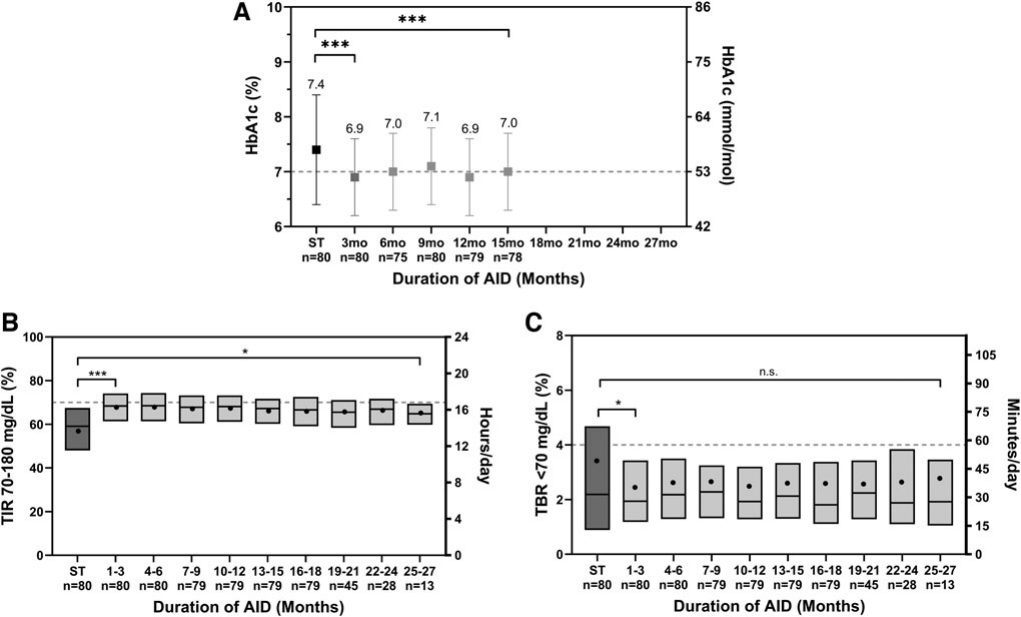
Glycemic outcomes during the standard therapy phase (ST), pivotal phase, and extension phase in 3-month intervals. **(A)** Mean HbA1c, **(B)** percentage TIR 70–180 mg/dL, and **(C)** percentage TBR (<70 mg/dL) are plotted in 3-month intervals for participants with data available. **(A)** Error bars show the standard deviation. **(B, C)** Box plots represent the median (line) with interquartile range (box) and mean (dots). *P < 0.05, ***P < 0.001, n.s., not significant with *P* ≥ 0.05. AID, automated insulin delivery; TBR, time below range; TIR, time in range.

HbA1c was reduced by 0.4% ± 0.7% (4.4 ± 7.7 mmol/mol, *P* < 0.0001), decreasing from 7.4% ± 1.0% (57 ± 10.9 mmol/mol) at baseline to 7.0% ± 0.7% (53 ± 7.7 mmol/mol) following 15 months of AID use (Fig. 1A and Supplementary Table S3). Improvement was seen regardless of baseline HbA1c, with those in the cohort with baseline HbA1c ≥8% (≥64 mmol/mol) maintaining a durable 0.9% ± 0.8% (9.8 ± 8.7 mmol/mol) improvement after 15 months (*P* < 0.0001) (Supplementary Fig. S1). Participants meeting the consensus target of HbA1c <7.0% (<53 mmol/mol) increased from 31% of children at baseline to 45% of children at the end of the extension phase.

From standard therapy to the extension phase, overall TIR increased by 10.0% ± 10.1% (57.2% ± 15.3% to 67.2% ± 9.3%, *P* < 0.0001) (Table 2), which corresponds to an increase of 2.4 h/day in range (70–180 mg/dL). This improvement was sustained from the pivotal study, with only a minor decrease in TIR of 0.9% ± 4.2% (*P* = 0.01) from pivotal to extension. To assess the stability of TIR over time, this outcome was plotted in 3-month intervals in Figure 1B. There was a noticeable drop in sample size between months 16–18, months 19–21, months 22–24, and months 25–27 from *n* = 79 to *n* = 45 to *n* = 28 to *n* = 13, corresponding to the time period when participants who had reached age 6 years or older were able to transition out of the study upon FDA clearance for those 6 years of age and older. Still, the improvement in TIR was stable over time when measured in 3-month intervals up to 25–27 months for children <6 years old who remained in the study for up to 2 years, as displayed in Figure 1B.

**Table 2.** Extension Study Results Overall for Children (Ages 2.0–5.9 Years), *N* = 80

Parameter	Standard therapy phase (14 days)	Pivotal study (3 months)	Extension study (16.7 ± 3.2 months)	Change from ST to pivotal study	*P*-value ST vs. pivotal	Change from ST to extension study	*P*-value ST vs. extension	Change from pivotal to extension	*P*-value pivotal vs. extension
Overall (24 h)									
Percentage time 70–180 mg/dL, %	57.2 ± 15.3, 59.1 (48.0, 67.5)	68.1 ± 9.0, 68.4 (61.4, 74.1)	67.2 ± 9.3, 67.4 (60.4, 72.8)	10.9 ± 9.6, 8.9 (4.9, 13.8)	<0.0001^a^	10.0 ± 10.1, 8.0 (2.9, 14.5)	<0.0001^a^	−0.9 ± 4.2, −1.1 (−3.0, 1.1)	0.0137^a^
Mean sensor glucose, mg/dL	171 ± 31, 164 (149, 189)	157 ± 17, 155 (147, 171)	159 ± 18, 161 (148, 172)	−14 ± 20, −10 (−18, −1)	<0.0001^a^	−12 ± 20, −9 (−19, 0)	<0.0001^a^	2 ± 8, 1 (−2, 5)	0.0315^a^
SD of sensor glucose, mg/dL	65 ± 13, 64 (56, 73)	60 ± 10, 60 (53, 66)	61 ± 11, 60 (55, 68)	−5 ± 8, −5 (−9, −1)	<0.0001^a^	−4 ± 8, −3 (−8, 2)	0.0001^a^	1 ± 4, 1 (−1, 3)	0.0036^a^
Coefficient of variation of sensor glucose, %^b^	38.1 ± 5.5, 37.4 (35.1, 41.7)	37.7 ± 4.0, 37.7 (35.1, 40.5)	38.1 ± 3.9, 37.5 (35.2, 41.1)	−0.4 ± 4.2, −0.5 (−3.6, 2.3)	0.4223^c^	0.0 ± 4.6, −0.1 (−2.9, 3.5)	0.9740^c^	0.4 ± 1.8, 0.5 (−0.6, 1.5)	0.0762^c^
Percentage time in glucose range, %									
<54 mg/dL	0.81 ± 1.68, 0.24 (0.05, 0.84)	0.47 ± 0.54, 0.26 (0.16, 0.60)	0.57 ± 0.60, 0.36 (0.20, 0.66)	−0.34 ± 1.33, 0.06 (−0.30, 0.16)	0.9394^a^	−0.24 ± 1.34, 0.10 (−0.15, 0.25)	0.2643^a^	0.10 ± 0.28, 0.05 (−0.01, 0.20)	<0.0001^a^
<70 mg/dL	3.43 ± 3.87, 2.19 (0.89, 4.68)	2.46 ± 1.83, 1.94 (1.18, 3.43)	2.61 ± 1.82, 2.13 (1.25, 3.01)	−0.97 ± 2.75, −0.27 (−1.54, 0.46)	0.0204^a^	−0.82 ± 2.81, 0.00 (−1.36, 0.61)	0.1355^a^	0.15 ± 0.81, 0.06 (−0.28, 0.68)	0.1080^c^
>180 mg/dL	39.4 ± 16.7, 37.0 (27.4, 50.0)	29.5 ± 9.8, 29.3 (23.1, 37.2)	30.2 ± 10.1, 31.6 (24.7, 37.5)	−9.9 ± 10.5, −7.6 (−12.8, ‑3.4)	<0.0001^a^	−9.2 ± 10.9, −8.0 (−12.6, −1.4)	<0.0001^a^	0.7 ± 4.4, 0.7 (−1.3, 3.0)	0.0603^a^
≥250 mg/dL	14.8 ± 12.1, 11.5 (5.4, 21.0)	9.2 ± 5.6, 8.4 (5.2, 13.0)	9.9 ± 6.0, 8.7 (5.7, 14.0)	−5.6 ± 8.9, ‑2.3 (‑6.6, ‑0.1)	<0.0001^a^	−4.9 ± 8.9, −2.7 (−7.6, 0.9)	<0.0001^a^	0.7 ± 2.8, 0.6 (−0.5, 1.5)	0.0045^a^
≥300 mg/dL	6.0 ± 7.3, 3.5 (1.1, 8.3)	3.2 ± 2.8, 2.4 (1.2, 4.6)	3.7 ± 3.2, 2.6 (1.5, 5.5)	−2.7 ± 6.1, −0.7 (−2.5, 0.2)	<0.0001^a^	−2.2 ± 5.9, −0.5 (−2.6, 0.7)	0.0033^a^	0.5 ± 1.8, 0.2 (−0.1, 0.8)	0.0009^a^
GRI	52 ± 21, 49 (39, 65)	37 ± 11, 36 (29, 45)	39 ± 11, 37 (31, 47)	−15 ± 14, −11 (−20, −5)	<0.0001^a^	−13 ± 14, −10 (−21, −4)	<0.0001^a^	2 ± 5, 2 (−1, 4)	0.0012^a^

Data are mean ± SD, median (IQR). To convert the values for glucose to millimoles per liter, multiply by 0.05551.

^a^ *P*-value determined using two-sided Wilcoxon signed-rank test.

^b^ Coefficient of variation of sensor glucose is SD divided by the mean.

^c^ *P*-value determined using unadjusted two-sided paired *t*-tests.

GRI, glycemia risk index; IQR, interquartile range; SD, standard deviation; ST, standard therapy.

Examination of TIR specifically during nighttime (00:00–06:00) revealed even greater improvement from standard therapy to the extension phase. Overnight, children experienced an increase of 20.0% ± 15.0% for TIR from standard therapy to extension (58.2% ± 18.7% to 78.2% ± 10.4%, *P* < 0.0001) (Table 3), amounting to nearly 1.2 h nightly.

**Table 3.** Extension Study Results During the Daytime and Overnight for Children (Ages 2.0–5.9 Years), *N* = 80

Parameter	Standard therapy phase (14 days)	Pivotal study (3 months)	Extension study (16.7 ± 3.2 months)	Change from ST to pivotal study	*P*-value ST vs. pivotal	Change from ST to extension study	*P*-value ST vs. extension	Change from pivotal to extension	*P*-value pivotal vs. extension
Daytime (06:00–24:00)									
Percentage time 70–180 mg/dL, %	56.9 ± 15.2, 58.2 (48.2, 68.1)	63.7 ± 9.7, 64.3 (55.9, 70.5)	63.5 ± 9.8, 63.4 (56.2, 69.5)	6.8 ± 9.7, 4.7 (1.3, 9.2)	<0.0001^a^	6.6 ± 10.3, 4.5 (−0.5, 10.1)	<0.0001^a^	−0.2 ± 4.6, 0.1 (−3.2, 1.9)	0.6209^a^
Mean sensor glucose, mg/dL	172 ± 31, 166 (152, 191)	163 ± 18, 162 (152, 177)	164 ± 19, 164 (152, 177)	−9 ± 21, −3 (−14, 2)	0.0005^a^	−9 ± 21, −5 (−15, 4)	0.0012^a^	1 ± 9, 0 (−4, 5)	0.6932^a^
SD of sensor glucose, mg/dL	66 ± 14, 67 (55, 77)	62 ± 11, 63 (56, 69)	63 ± 11, 62 (57, 72)	−4 ± 8, −3 (‑8, 2)	<0.0001^a^	−3 ± 8, −1 (−7, 3)	0.0344^a^	1 ± 4, 1 (−1, 4)	0.0136^b^
Coefficient of variation of sensor glucose, %^c^	38.6 ± 5.7, 38.1 (34.9, 42.9)	38.0 ± 3.8, 37.8 (35.9, 40.7)	38.6 ± 3.7, 38.1 (36.3, 41.5)	−0.6 ± 4.1, −0.2 (−3.1, 1.8)	0.2249^b^	0.0 ± 4.5, 0.1 (−2.1, 2.9)	0.9879^b^	0.6 ± 1.8, 0.6 (−0.5, 1.8)	0.0074^b^
Percentage time in glucose range, %									
<54 mg/dL	0.80 ± 1.67, 0.16 (0.00, 0.73)	0.50 ± 0.61, 0.29 (0.16, 0.62)	0.59 ± 0.63, 0.38 (0.22, 0.65)	−0.29 ± 1.24, 0.09 (−0.19, 0.16)	0.5395^a^	−0.20 ± 1.28, 0.12 (−0.10, 0.30)	0.1686^a^	0.09 ± 0.28, 0.05 (−0.03, 0.21)	0.0003^a^
<70 mg/dL	3.44 ± 3.85, 2.03 (0.83, 4.42)	2.57 ± 1.99, 1.98 (1.27, 3.35)	2.79 ± 1.98, 2.29 (1.37, 3.30)	−0.87 ± 2.53, 0.00 (−1.61, 0.42)	0.0799^a^	−0.65 ± 2.64, 0.09 (−1.44, 0.90)	0.3100^a^	0.22 ± 0.88, 0.15 (−0.30, 0.74)	0.0318^b^
>180 mg/dL	39.7 ± 16.6, 37.2 (28.0, 50.2)	33.7 ± 10.7, 33.2 (27.6, 42.4)	33.7 ± 10.8, 33.9 (27.6, 41.6)	−6.0 ± 10.5, −3.2 (−8.2, 0.0)	<0.0001^a^	−6.0 ± 11.0, −3.5 (−9.8, 1.0)	<0.0001^a^	0.0 ± 4.9, −0.3 (−2.7, 3.4)	0.9356^a^
≥250 mg/dL	15.4 ± 12.7, 11.9 (6.1, 21.8)	11.0 ± 6.6, 9.9 (6.2, 15.4)	11.5 ± 6.9, 10.3 (7.0, 16.3)	−4.4 ± 9.2, −1.3 (−5.4, 0.6)	<0.0001^a^	−3.9 ± 9.3, −1.4 (−5.8, 1.4)	0.0008^a^	0.5 ± 3.4, 0.2 (−0.9, 1.5)	0.1220^a^
≥300 mg/dL	6.5 ± 8.0, 4.2 (1.3, 9.2)	3.9 ± 3.4, 3.0 (1.5, 5.6)	4.4 ± 3.8, 3.1 (1.8, 6.6)	−2.6 ± 6.7, −0.6 (−2.8, 0.6)	0.0018^a^	−2.1 ± 6.3, −0.3 (−2.3, 0.9)	0.0264^a^	0.5 ± 2.1, 0.3 (−0.2, 0.7)	0.0135^a^
Overnight (00:00–06:00)									
Percentage time 70–180 mg/dL, %	58.2 ± 18.7, 60.6 (47.8, 70.1)	81.0 ± 10.0, 82.4 (76.8, 88.7)	78.2 ± 10.4, 80.1 (70.6, 85.9)	22.8 ± 14.8, 19.5 (12.8, 32.2)	<0.0001^a^	20.0 ± 15.0, 18.2 (9.8, 28.8)	<0.0001^b^	−2.8 ± 5.1, −3.6 (−6.7, 0.9)	<0.0001^b^
Mean sensor glucose, mg/dL	168 ± 33, 164 (148, 189)	141 ± 16, 141 (129, 150)	146 ± 17, 145 (132, 159)	−27 ± 25, −23 (−45, −9)	<0.0001^a^	−22 ± 26, −19 (−39, −4)	<0.0001^b^	5 ± 8, 4 (−1, 10)	<0.0001^b^
SD of sensor glucose, mg/dL	58 ± 14, 58 (50, 64)	46 ± 11, 46 (37, 52)	49 ± 12, 47 (40, 55)	−13 ± 12, −11 (−21, −7)	<0.0001^b^	−9 ± 12, −9 (−16, −2)	<0.0001^b^	3 ± 6, 3 (−1, 7)	<0.0001^b^
Coefficient of variation of sensor glucose, %^c^	34.7 ± 6.6, 35.2 (30.9, 38.8)	32.1 ± 5.2, 31.6 (29.2, 35.3)	33.2 ± 5.1, 33.6 (30.5, 36.1)	−2.6 ± 6.7, ‑3.6 (‑6.9, −0.3)	0.0002^a^	−1.5 ± 6.6, −2.0 (−5.6, 2.8)	0.0407^b^	1.1 ± 3.3, 1.5 (−1.1, 3.6)	0.0051^b^
Percentage time in glucose range, %									
<54 mg/dL	0.85 ± 1.94, 0.00 (0.00, 0.97)	0.39 ± 0.53, 0.18 (0.06, 0.53)	0.49 ± 0.63, 0.27 (0.13, 0.61)	−0.46 ± 1.78, 0.00 (−0.51, 0.13)	0.1141^a^	−0.36 ± 1.75, 0.07 (−0.47, 0.25)	0.8530^a^	0.11 ± 0.43, 0.08 (−0.04, 0.23)	0.0031^a^
<70 mg/dL	3.41 ± 4.79, 1.66 (0.40, 4.21)	2.13 ± 1.94, 1.58 (0.65, 2.89)	2.06 ± 1.70, 1.46 (0.84, 2.91)	−1.28 ± 4.17, −0.44 (−2.23, 0.63)	0.0185^a^	−1.35 ± 4.18, −0.35 (−2.13, 0.79)	0.0344^a^	−0.07 ± 1.12, 0.14 (−0.62, 0.44)	0.8196^a^
>180 mg/dL	38.4 ± 20.1, 36.5 (24.8, 51.1)	16.9 ± 10.3, 15.5 (8.4, 21.8)	19.7 ± 10.6, 18.1 (12.3, 28.3)	−21.5 ± 16.0, −19.1 (−31.5, ‑11.3)	<0.0001^b^	−18.7 ± 16.3, −17.2 (−28.7, −7.7)	<0.0001^b^	2.9 ± 5.1, 3.4 (−0.7, 6.2)	<0.0001^b^
≥250 mg/dL	13.0 ± 13.2, 8.3 (3.4, 17.6)	3.9 ± 3.9, 3.1 (1.2, 5.0)	5.1 ± 4.6, 3.3 (1.8, 7.7)	−9.1 ± 11.4, ‑5.1 (‑13.8, ‑1.0)	<0.0001^a^	−7.8 ± 11.2, −4.0 (−13.2, 0.0)	<0.0001^a^	1.2 ± 2.2, 0.9 (−0.2, 2.7)	<0.0001^a^
≥300 mg/dL	4.3 ± 6.7, 1.3 (0.0, 5.6)	1.2 ± 1.6, 0.6 (0.1, 1.9)	1.8 ± 2.1, 0.9 (0.3, 2.5)	−3.1 ± 6.1, −0.6 (−4.7, 0.0)	<0.0001^a^	−2.5 ± 6.0, 0.0 (−4.2, 0.4)	0.0058^a^	0.6 ± 1.3, 0.4 (0.0, 1.0)	<0.0001^a^

Data are mean ± SD, median (IQR). To convert the values for glucose to millimoles per liter, multiply by 0.05551.

^a^ *P*-value determined using two-sided Wilcoxon signed-rank test.

^b^ *P*-value determined using unadjusted two-sided paired *t*-tests.

^c^ Coefficient of variation of sensor glucose is SD divided by the mean.

IQR, interquartile range; SD, standard deviation; ST, standard therapy.

Secondary outcomes revealed that the time spent in hypoglycemia remained stable, with no change in percentage of time <70 mg/dL (*P* > 0.05) as well as no change in time <54 mg/dL (*P* > 0.05) from standard therapy to extension. Most participants had a low percentage of time <70 mg/dL with standard therapy, with a median of 2.19% (27.5% of participants with <1% of time <70 mg/dL at baseline); however, the higher mean value of 3.43% indicates some outliers. While the median percentage of time <70 mg/dL remained relatively stable over time (Fig. 1C), there was a more apparent decrease in the mean value from standard therapy to the pivotal phase, where six participants (6/80, 7.5%) saw a decrease of ≥5%. Overnight, the percentage of time <70 mg/dL decreased by median [IQR] 0.35% [−2.13, 0.79] from 1.66% [0.40, 4.21] with standard therapy to 1.46% [0.84, 2.91] during the extension (*P* = 0.03). Time spent in hyperglycemia was reduced as shown by a decrease in time >180 mg/dL by 9.2% ± 10.9% from 39.4% ± 16.7% with standard therapy to 30.2% ± 10.1% (*P* < 0.0001) during the extension.

These secondary outcomes were largely maintained from the original 3-month pivotal study, with a minor increase in time <54 mg/dL (*P* < 0.0001), a minor increase in time ≥250 mg/dL (*P* = 0.005), and a minor increase in time ≥300 mg/dL (*P* = 0.0009) from the pivotal to extension phase. An exploratory analysis of the glycemia risk index^13^ showed that this measure decreased from 52 ± 21 with standard therapy to 37 ± 11 during the pivotal phase and 39 ± 11 during the extension, with a slight increase from pivotal to extension (Table 2).

**FIG. 2.**
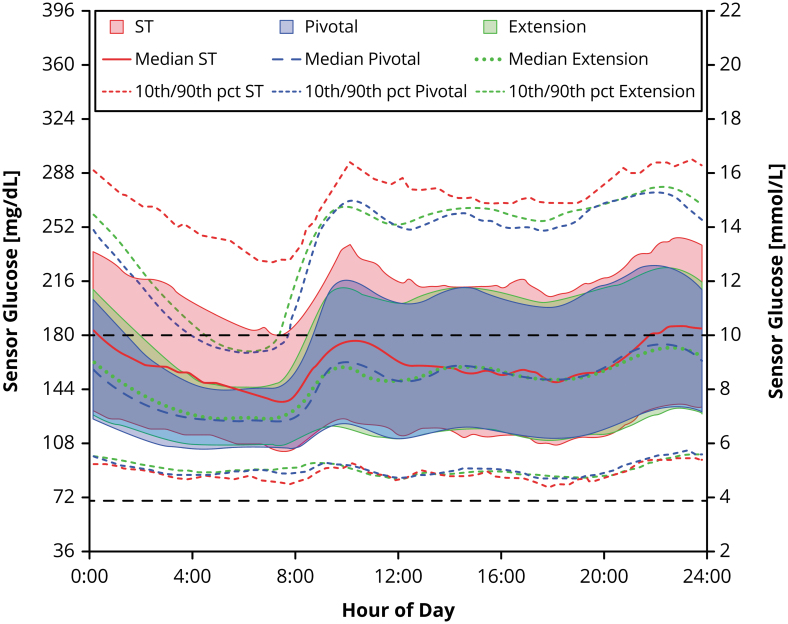
Sensor glucose measurements. Median sensor glucose measurement for children (age 2.0–5.9 years, *n* = 80) during the standard therapy (ST) phase (red line), pivotal phase (blue line), and extension phase (green line), with shaded areas indicating the interquartile range for each phase. The target range (70–180 mg/dL) is indicated by the black dashed lines. Measurements represent a 24-h period from midnight to midnight.

Mean sensor glucose was reduced by 12 ± 20 mg/dL (*P* < 0.0001) in the extension phase compared with standard therapy (Table 2), with a minor increase in mean glucose of 2 ± 8 mg/dL (*P* = 0.03) from pivotal to extension phase. A profile of sensor glucose measurements, evaluated by time of day, can be seen in Figure 2 for all three phases (standard therapy, pivotal, and extension). This profile highlights the change in sensor glucose overall from standard therapy to extension and emphasizes the overnight results that revealed a decrease in mean glucose of 22 ± 26 mg/dL (*P* < 0.0001), as compared with the decrease of 9 ± 21 mg/dL (*P* = 0.001) during the daytime.

**FIG. 3.**
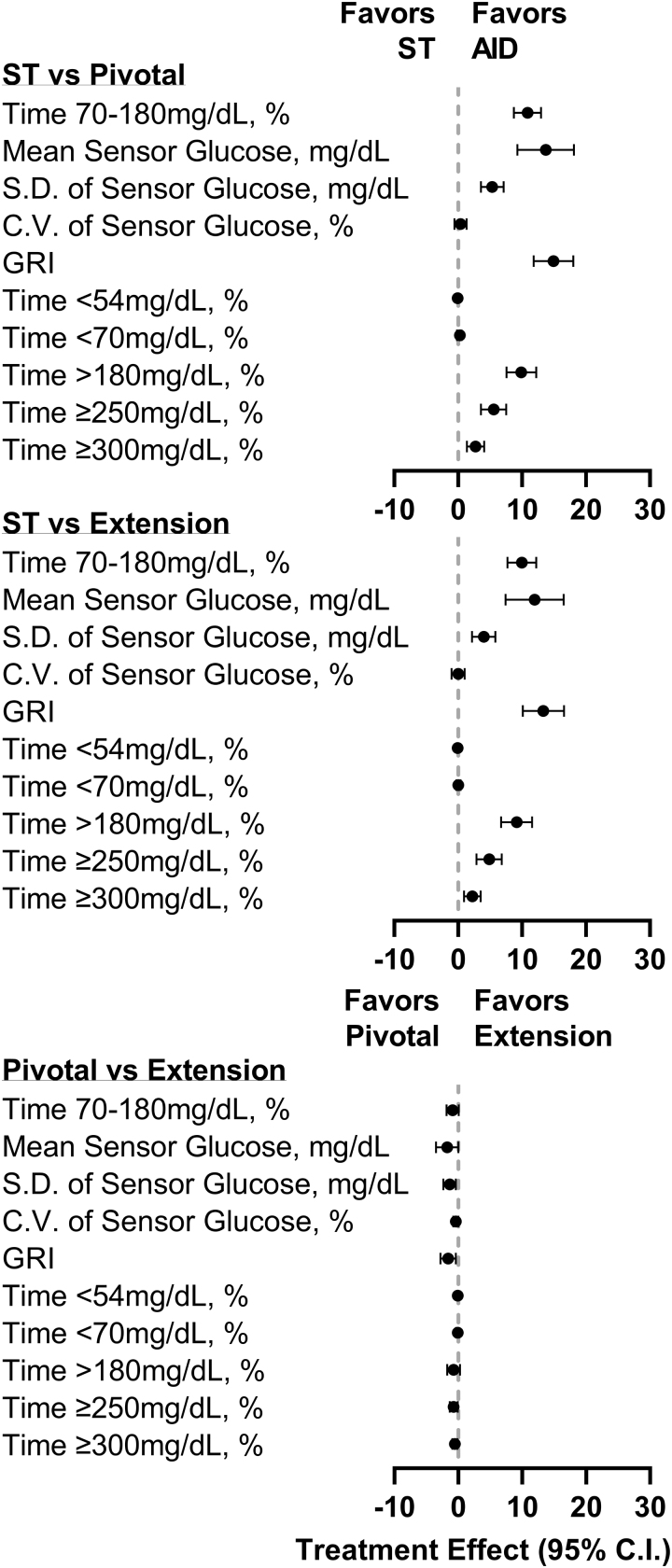
Treatment effect on CGM metrics. Forest plot of CGM outcomes during the standard therapy (ST) phase, pivotal phase, and extension phase. Treatment effect for each metric was calculated such that a positive treatment effect indicated an improvement. Time <54 and <70 mg/dL are shown as median with the 95% confidence interval. All other data are shown as mean with the 95% confidence interval. AID, automated insulin delivery; CGM, continuous glucose monitor; CV, coefficient of variation; GRI, glycemia risk index; SD, standard deviation.

A visual comparison of the change in all glycemic outcomes across the three phases is depicted in Figure 3. Select outcomes stratified by standard therapy method (MDI or pump) are presented in Supplementary Table S4. The number of participants meeting established clinical targets^14^ for glycemia during each phase is shown in Supplementary Table S5, with 78% achieving >60% TIR during the extension phase as compared with 46% achieving >60% TIR during standard therapy. The percentage of participants meeting the composite target of >70% TIR and <4% of time <70 mg/dL was 26% at extension, compared with 11% during standard therapy.

### Safety outcomes

Throughout the extension phase, the occurrence of severe hypoglycemia and diabetic ketoacidosis was 0.90 and 0.90 events per 100 person-years, respectively (Supplementary Table S6). One severe hypoglycemia event occurred, which was deemed unrelated to the study device, following emesis after a user-initiated bolus without eating. One instance of diabetic ketoacidosis was recorded, also deemed unrelated to the study device, which progressed from a viral infection. There were 52 total instances of hyperglycemia with ketosis (blood glucose meter ≥300 mg/dL and ketones >1.0 mmol/L), affecting 32.5% of the participants during the extension phase, corresponding to a rate of 46.7 events per 100 person-years. Of these events, 23 were deemed possibly related and 20 were deemed related to the study device. A more detailed list of all adverse events reported during the extension phase is viewable in Supplementary Table S6.

### System use

While in the extension phase, participants spent median [IQR] 97.1% [94.8, 97.9] of time in Automated Mode (Supplementary Table S7). Time in Automated Mode was generally stable over time, with some variability possibly attributable to the changing sample size and other transient events. Roughly 0.36 device deficiencies per person-month of extension system use occurred: 72.2% related to the Pod, 14.8% related to the Omnipod 5 App as a controller, 9.6% related to the CGM transmitter, 3.1% related to the CGM sensor, and 0.2% related to the ketone meter.

### Insulin and body weight

Total daily insulin requirements did not reveal a significant difference from 0.69 ± 0.18 U/kg with standard therapy to 0.71 ± 0.15 U/kg (*P* > 0.05) during the pivotal phase but did reveal an increase to 0.80 ± 0.17 U/kg (*P* < 0.0001) during the extension phase (Supplementary Table S8). The percentage of total daily insulin from user-initiated boluses decreased from 58.7% ± 13.6% during standard therapy to 53.5% ± 8.1% during the extension phase (*P* = 0.0001) (Supplementary Table S8).

There was no significant change in BMI or BMI z-score between baseline, 3 months, and 15 months of use, indicating maintenance of growth patterns (Supplementary Table S8).

## Discussion

This trial demonstrated the safety and effectiveness of the Omnipod 5 AID System in very young children with type 1 diabetes, as 99% of participants (79/80) opted to continue use through the full duration of the extension phase (up to 2 years of at home use). The long-term durability of the system effectiveness was evident by the sustained improvements in glycemic outcomes through the extension phase.

This cohort of very young children spent 2.4 more hours per day in target range, with an overall increase of 10.0% ± 10.1% TIR from standard therapy to the extension phase. Overnight, children experienced an even greater increase in TIR of 20.0% ± 15.0% from standard therapy to extension. Improvements in HbA1c seen in the pivotal trial were also sustained, settling at 7.0% ± 0.7% (53 ± 7.7 mmol/mol) at 15 months (no further measurements were taken). The percentage of children achieving the consensus target of HbA1c <7% (<53 mmol/mol) increased from 31% to 45% from baseline to 15 months. These glycemic improvements were sustained with a very low rate of severe hypoglycemia and diabetic ketoacidosis, and a low percentage of time <70 mg/dL both overall and overnight. The number of participants achieving either >60% TIR or >70% TIR along with <4% of time <70 mg/dL approximately doubled from standard therapy to extension.

Data on the use of AID systems for very young children are limited, particularly when it comes to sustained use over a long period of time. This is the longest study, with up to 24.7 total months of system use, for a study group of this age range published to date, with glycemic outcomes consistent with those reported for other AID studies in very young children using systems for shorter durations (3–7 months). It should be noted that although these studies all included the same age range, the actual mean age of participants enrolled was variable and may limit comparisons that can be made between them. Wadwa et al. reported the use of another AID system in a randomized controlled trial in very young children (2 to <6 years) with a 3-month extension phase.^5,8^ In the group using the AID system, TIR increased from 56.7% ± 18.0% at baseline to 69.3% ± 11.1% during the 3-month study,^8^ with the increase maintained during a 3-month extension phase.^5^ The percentage of participants in that study meeting >70% TIR and <4% time below range was 31%, similar to that in the present study (26%).

Three other studies of AID use in this age group that reported outcomes from a shorter duration demonstrated an improvement in TIR of ∼8.1%–8.7% over a period of ∼3–4 months.^6,7,9^ A recent real-world study of another AID system in very young children over a 7-month period reported a slightly lower TIR of 66.9% ± 11.7% than what has been reported for this current study, as well as a somewhat greater percent time below 70 mg/dL of median 3.0% [1.8, 4.5]; however, real-world results are not directly comparable to those from a clinical trial.^10^ Thus, results reported in this current study using Omnipod 5 show comparable improvements with other AID systems for this age group in clinical trials and real-world studies, but over a substantially longer duration of use.

A particular strength of this study is the length of time over which glycemic outcomes for participants were monitored, namely for up to 2 years, with 25% of participants using the system for at least 2 years of total use. This long observation period is especially salient given the recognition that young children are experiencing rapid physical growth while undergoing many developmental changes, such as starting preschool. This combination of changes in young children with type 1 diabetes generally leads to many adjustments to the diabetes management plan. Thus, the ability of an AID system, in use for up to 2 years, to manage the multitude of changes in these young children is particularly meaningful. Furthermore, there are few studies assessing the impact of AID systems in very young children and all are shorter in duration.

This extension phase also incorporated fewer study visits as compared with the pivotal phase, likely resulting in a decrease in “study effect” on glycemic outcomes that may have occurred during initial study use with more frequent study visits. However, the frequency of follow-up visits throughout the extension phase was still greater than routine observation outside of a clinical study and thus the results may not be generalizable in a real-world setting.

A notable limitation of this study is the single-arm design, as the possible impact of patient selection and study interactions may have played a role in glycemic improvements, which could not be accounted for without a control group. While this study did target a specific sample of young children, there was a lack of diversity in ethnicity (77.5% white, not Hispanic or Latino) and baseline insulin regimen (85% with previous/current pump use) making results less generalizable across various groups in this age range. However, previous pump use was not a requirement for study enrollment, allowing for a subset of users who have not experienced pump technology.

## Conclusion

Few studies have evaluated the impact of AID systems on very young children with type 1 diabetes in the early stages of their development. The Omnipod 5 AID System was previously shown to be safe and effective in very young children ages 2–5.9 years; however, data on the durability of these results are limited. This extension study highlights the long-term benefits of the Omnipod 5 AID System in very young children with sustained outcomes for up to a 2-year period that were initially seen in the 3-month pivotal study phase. These findings provide the longest prospective follow-up data on children in this age group initiating an AID system. The observed glycemic improvements overall and specifically overnight will have significant effects in reducing diabetes-related complications, especially vital when considering this population that will live with diabetes for an extensive period of time.

## Supplementary Material

Supplementary Appendix SA1
