# Supplementary material for: Glycemic Outcomes Persist for up to 2 Years in Very Young Children with the Omnipod® 5 Automated Insulin Delivery System
Source: Diabetes Technol Ther. 2024 Jun 30;26(6):383–93. doi: 10.1089/dia.2023.0506 (PMC12776363; doi:10.1089/dia.2023.0506)
Supplement: Supplementary Appendix SA1 [file dia.2023.0506_suppl_data.pdf]

SUPPLEMENTARY APPENDIX

This appendix has been provided by the authors to give readers additional information about their work.

Supplement to: Glycemic Outcomes Persist for up to 2 Years in Very Young Children with the Omnipod® 5 Automated Insulin Delivery System

Contents

SUPPLEMENTARY APPENDIX 1

Omnipod 5 in Preschoolers Study Group 2

Table S1. Eligibility and exclusion criteria 4

Table S2. Schedule of participant visits 5

Table S3. HbA1c results over time for participants electing to participate in the extension phase 7

Table S4. Mean glycemic outcomes for the extension study at baseline or standard therapy and the extension study phase, stratified by baseline characteristics 8

Table S5. Number of participants meeting consensus targets[1] for glycemic control during the standard therapy phase and the extension phase (N=80) 9

Table S6. Safety outcomes during extension phase[*] 10

Table S7. Percentage of time (%) in Automated Mode in 3-month intervals during the pivotal phase and extension phase 12

Table S8. Insulin requirements and weight changes during the standard therapy, pivotal, and extension phases 13

Supplementary Figure S1. Subgroup analysis of HbA1c in 3-month intervals stratified by baseline HbA1c. 15

REFERENCES 16

Omnipod 5 in Preschoolers Study Group
Clinical site principal investigators (P.I.) are noted.

Department of Pediatrics, Baylor College of Medicine, Houston, TX
Daniel J. DeSalvo MD (P.I.), Siripoom McKay (Co-I), Kylie DeLaO, RN, CDCES (Study Nurse), Carolina Villegas, MS (Study Coordinator)

Department of Pediatrics, Yale School of Medicine, New Haven, CT
Jennifer L. Sherr MD, PhD (P.I.), Kate Weyman, APRN, FNP-C, CDE (Co-I), Eileen-Tichy, PA-C (Co-I), Michelle VanName, MD (Co-I), Michelle Brei, APRN, DNP (Co-I), Melinda Zgorski, BSN (Study Nurse), Amy Steffen, BSN (Study Nurse), Lori Carria, MS (Study Coordinator)

Atlanta Diabetes Associates, Atlanta GA
Bruce W. Bode (P.I.), Anna Busby RD CDE

Barbara Davis Center for Diabetes, University of Colorado Anschutz Medical Campus, Aurora, CO
Gregory P. Forlenza MD (P.I.), R. Paul Wadwa MD (Co-I), Robert Slover MD (Co-I), Erin Cobry MD (Co-I), Cari Berget RN, MPH, CDCES (lead study nurse), Laurel Messer RN, CDCES, PhD (study nurse)

Joslin Diabetes Center, Harvard Medical School, Boston, MA
Lori M. Laffel MD MPH (P.I.), Elvira Isganaitis MD MPH (Co-I), Louise Ambler-Osborn PNP (Study Nurse), Emily Freiner FNP (Study Nurse), Christine Turcotte PNP (Study Nurse), Lisa Volkening MA CCRP (Regulatory Coordinator)

Division of Endocrinology, Center for Diabetes Technology, University of Virginia, Charlottesville, VA
Melissa Schoelwer MD (Co-I), Sue A. Brown MD (P.I.), Katie Krauthause (Research Coordinator), Emma Emory, RN (Research Coordinator), Mary Oliveri (Regulatory Coordinator)

Department of Pediatrics, Division of Pediatric Endocrinology, Stanford University, Stanford, CA
Bruce A. Buckingham MD (P.I.), Laya Ekhlaspour (Co-I), Ryan Kingman (Study Coordinator).

International Diabetes Center, Park Nicollet Pediatric Endocrinology, Minneapolis, MN
Amy B. Criego (P.I.), Betsy L. Schwartz (sub-investigator), Laura M. Gandrud (sub-investigator), Aimee Grieme (lead study coordinator), Jamie Hyatt (study coordinator)

Department of Pediatrics, University Hospitals Cleveland Medical Center, Rainbow Babies and Children's Hospital, Cleveland, OH
Sarah A. MacLeish DO (P.I.), Jamie R. Wood MD (co-investigator), Beth A. Kaminski MD (co-investigator), Terri Casey, RN, BSN, CDCES (lead study coordinator), Wendy Campbell RN, BSN, CDCES (study nurse), Kim Behm, RN BSN, CDCES (study nurse), Ramon Adams CCRP (regulatory coordinator)

Department of Pediatrics, SUNY Upstate Medical University, Syracuse, NY
David W. Hansen MD MPH (P.I.), Sheri L. Stone, MSN, RN, FNP-C (sub-investigator), Suzan Bzdick, RN, CDCES, CCRC (Lead Coordinator/Study Nurse), Jane Bulger, MS, CCRC (study coordinator), Lynn Agostini, AAS, CCRP (study coordinator), Sarah Doolittle, BS, CCRC (regulatory coordinator)

Labcorp, Burlington, NC
Kaisa Kivilaid MS (Lead Biostatistician), Krista Kleve MS (Project Manager)

Insulet Corporation, Acton, MA

Trang T. Ly MBBS, Bonnie Dumais RN, Todd Vienneau BSc, Lauren M. Huyett PhD, Lindsey R. Conroy PhD, Joon Bok Lee PhD, Jason O'Connor BSME, Eric Benjamin M.Eng., MBA

Table S1. Eligibility and exclusion criteria

Inclusion Criteria	Subjects must meet all of the following criteria in order to be enrolled in the study:<br>1. Age at time of consent 2.0-5.9 years.<br>2. Living with parent/legal guardian.<br>3. Diagnosed with type 1 diabetes. Diagnosis is based on investigator's clinical judgment.<br>4. Deemed appropriate for pump therapy per investigator's assessment taking into account previous history of severe hypoglycemic and hyperglycemic events, and other comorbidities.<br>5. Investigator has confidence that the subject and/or parent/guardian/caregiver can successfully operate all study devices and is capable of adhering to the protocol.<br>6. Willing to use only the following types of insulin during the study: Humalog, Novolog, Admelog or Apidra during the study.<br>7. Willing to wear the system continuously throughout the study.<br>8. Willing to participate in challenges for 2 consecutive days, consisting of a minimum of 3 hours of activity per day and dietary challenge by reducing the number of carbohydrates entered for a given meal by 50%.<br>9. HbA1c <10% at screening visit.<br>10. Subject and/or parent/guardian must be willing to use the Dexcom App on the Omnipod Horizon™ PDM as the sole source of Dexcom data (with the exception of the Dexcom Follow App) during the automated insulin delivery phase.<br>11. Subject and/or parent/guardian is able to read and speak English fluently.<br>12. Has a parent/legal guardian willing and able to sign the informed consent form.
Exclusion Criteria	Subjects who meet any of the following criteria will be excluded from the study:<br>1. A medical condition, which in the opinion of the investigator, would put the subject at an unacceptable safety risk.<br>2. History of severe hypoglycemia in the past 6 months.<br>3. History of DKA in the past 6 months, unrelated to an intercurrent illness, infusion set failure or initial diagnosis.<br>4. Diagnosed with sickle cell disease.<br>5. Diagnosed with hemophilia or any other bleeding disorders.<br>6. Plans to receive blood transfusion over the course of the study.<br>7. Clinical evidence of acute or chronic kidney disease (e.g., estimated GFR < 45) or currently on hemodialysis.<br>8. History of adrenal insufficiency.<br>9. Has taken oral or injectable steroids within the past 8-weeks or plans to take oral or injectable steroids during the course of the study.<br>10. Unable to tolerate adhesive tape or has any unresolved skin condition in the area of sensor or pump placement.<br>11. Plans to use insulin other than U-100 insulin intended for use in the study device during the course of the study.<br>12. Use of non-insulin anti-diabetic medication other than metformin (e.g., GLP1 agonist, SGLT2 inhibitor, DPP-4 inhibitor, pramlintide).<br>13. Thyroid Stimulating Hormone (TSH) is outside of normal range with clinical signs of hypothyroidism or hyperthyroidism.<br>14. Currently participating or plans to participate in another clinical study using an investigational drug or device other than the Omnipod Horizon™ Automated Glucose Control System during this study period.<br>15. Unable to follow clinical protocol for the duration of the study or is otherwise deemed unacceptable to participate in the study per the investigator's clinical judgment.

Abbreviations: PDM=Personal Diabetes Manager, DKA=Diabetic Ketoacidosis, GFR= Glomerular Filtration Rate, GLP1=Glucagon-like peptide 1, SGLT2=Sodium-glucose cotransporter-2, DPP-4=Dipeptidyl peptidase 4.

Table S2. Schedule of participant visits

Assessment Schedule	Automated Insulin Delivery Phase (AID)												
	Extension Phase (Phase 3)												
Visit Number	14	15	16	17	18	19	20	21	22	23 or Study Exit	Extension Through Commercialization	UV	EW[§§]
Study Day/Visit Window[*]	120	150	180	210	240	270	315	360	405	450	± 45d ± 5d	-	-
	± 5d	± 5d	± 5d	± 5d	± 5d	± 5d	± 5d	± 5d	± 5d	± 5d	(or sooner if Study Exit)		
Telephone (T) or Office (O) Visit	T/O[l]	T/O[l]	T/O[l]	T/O[l]	T/O[l]	T/O[l]	T/O[l]	T/O[l]	T/O[l]	T/O[l]	T/O[l]	T/O[l]	O
**Laboratory Assessments**													
HbA1c			X			X		X		X			
**Clinical Assessments**													
Informed Consent						X[n]				X[n]			
Concomitant Medications	X	X	X	X	X	X	X	X	X	X	X	X	X
Height										X			X
Weight										X			X
Adverse Events	X	X	X	X	X	X	X	X	X	X	X	X	X
**Study Devices**													
Return Omnipod 5 AID System										X	X		X
Complaints/Device Deficiencies	X	X	X	X	X	X	X	X	X	X	X	X	X
Device Uploads (BG/Ketone meter)	X	X	X	X	X	X	X	X	X	X	X		X
Data Review[m]	X	X	X	X	X	X	X	X	X	X	X	X	X

Abbreviations: AID=Automated Insulin Delivery; EW=Early Withdrawal; UV=Unscheduled Visit, BG= Blood Glucose.

[l]Device uploads from the BG and Ketone meter are not required at any visit conducted via telephone.
[m]Data review for Automated Mode occur at all visits. Data review for BG/Ketone Meters to occur during onsite visits only.

[n]Subjects extending their participation into the extension phase are to be consented for both the first 6-month and the second 6-month extension,

as well as the extension through commercial availability. Consent for each interval must occur on or any time before commencing that study interval (e.g. consent for first 6-months of Phase 3 must occur on or before Visit 13, consent for second 6-months of Phase 3 must occur on or before Visit 19, consent for visits beyond Visit 23 until commercial availability must occur on or before Visit 23).

[O]Devices will be returned to the site at the end of the subject's participation if the subject is not participating in the extension phase.

[P]Extension Through Commercialization (ETC) visits begin with Visit 24 and each additional visit will be numbered in consecutive order ending with the Study Exit visit.

Table S3. HbA1c results over time for participants electing to participate in the extension phase

Parameter	Baseline	3 Months AID (Pivotal End)	6 Months AID	9 Months AID	12 Months AID	15 Months AID
N with HbA1c available	80	80	75	80	79	78
HbA1c (%, mmol/mol)	7.4 ± 1.0, 57 ± 10.9	6.9 ± 0.7, 52 ± 7.7	7.0 ± 0.7, 53 ± 7.7	7.1 ± 0.7, 54 ± 7.7	6.9 ± 0.7, 52 ± 7.7	7.0 ± 0.7, 53 ± 7.7
HbA1c (%, mmol/mol) change from baseline	---	-0.5 ± 0.6, -5.5 ± 6.6	-0.4 ± 0.6, -4.4 ± 6.6	-0.3 ± 0.6, -3.3 ± 6.6	-0.5 ± 0.6, -5.5 ± 6.6	-0.4 ± 0.7, -4.4 ± 7.7
P-value from baseline	---	<0.0001[2]	<0.0001[2]	<0.0001[2]	<0.0001[2]	<0.0001[2]
% with HbA1c <7%	25 (31%)	43 (54%)	34 (45%)	32 (40%)	38 (48%)	35 (45%)

Data are shown as mean ± S.D.

[1] Unadjusted two-sided p-value for paired t-test.
[2] Two-sided Wilcoxon signed rank test.

Table S4. Mean glycemic outcomes for the extension study at baseline or standard therapy and the extension study phase, stratified by baseline characteristics

	**% Time in range 70-180mg/dL**			**% Time below 70mg/dL§**			**% Time above 180mg/dL**			**HbA1c (%) [mmol/mol]**		
**Parameter**	Standard Therapy Phase	Extension Study	*p*-value	Standard Therapy Phase	Extension Study	*p*-value	Standard Therapy Phase	Extension Study	*p*-value	Baseline	Follow-up	*p*-value
**Overall (N=80)**	57.2±15.3	67.2±9.3	<0.0001[2]	2.19 (0.89, 4.68)	2.13 (1.25, 3.01)	0.1355[2]	39.4±16.7	30.2±10.1	<0.0001[2]	7.4±1.0 [57±10.9]	7.0±0.7 [53±7.7]	<0.0001[2]
**Prior Therapy Method**												
Multiple daily injections (n=12)	48.4±14.9	62.3±7.5	0.0061[1]	1.45 (0.45, 5.31)	1.66 (1.21, 2.76)	0.7910[2]	48.0±16.3	35.5±7.1	0.0276[1]	8.4±0.9 [68±9.8]	7.5±0.6 [58±6.6]	0.0050[1]
Pump (n=68)	58.8±15.0	68.0±9.3	<0.0001[2]	2.45 (1.01, 4.83)	2.16 (1.27, 3.12)	0.1330[2]	37.9±16.4	29.3±10.4	<0.0001[2]	7.3±0.9 [56±9.8]	7.0±0.7 [53±7.7]	<0.0001[2]

To convert the values for glucose to millimoles per liter, multiply by 0.05551.
[1]p-value determined using two-sided paired t-tests.
[2]p-value determined using two-sided Wilcoxon signed rank tests.
§Values presented for % Time below 70mg/dL are median (IQR). IQR denotes interquartile range. Remaining values in the table are mean ± SD.

Table S5. Number of participants meeting consensus targets[1] for glycemic control during the standard therapy phase and the extension phase (N=80)

Number of participants meeting target, n (%)	**Baseline † or Standard Therapy Phase**	**Follow-up † or Extension Phase**
HbA1c* <7.0% (<53 mmol/mol)	25 (31%)	35 (45%)
HbA1c* <7.5% (<58 mmol/mol)	42 (53%)	58 (74%)
Time in range 70-180mg/dL >60%	37 (46%)	62 (78%)
Time in range 70-180mg/dL >70%	14 (18%)	29 (36%)
Time <70mg/dL <4%	57 (71%)	66 (83%)
Composite – Time in range >60% and time <70mg/dL <4%	23 (29%)	49 (61%)
Composite – Time in range >70% and time <70mg/dL <4%	9 (11%)	21 (26%)

To convert the values for glucose to millimoles per liter, multiply by 0.05551.
*Baseline HbA1c values were available for 80 children. Final HbA1c values were available for 78 children at 15 total months of AID use.
†Baseline and follow-up (Visit 23) data were used for the primary effectiveness outcomes of HbA1c, the remaining outcomes are described for the standard therapy phase and the extension phase.

Table S6. Safety outcomes during extension phase*

Event Type	
**Primary Safety Outcomes (events per 100 person-years)**†	
Severe hypoglycemia	0.90
Diabetic ketoacidosis	0.90
Hypoglycemia, number of events (% of participants)‡	2 (2.5)
Severe Hypoglycemia, number of events (% of participants)§	1 (1.3)
Diabetic Ketoacidosis, number of events (% of participants)‖	1 (1.3)
Hyperglycemia, number of events (% of participants)¶	3 (3.8)
Prolonged Hyperglycemia, number of events (% of participants)#	52 (32.5)
Other, number of events (% of participants)**	53 (45)

*Extension phase was calculated from the end of the pivotal clinical trial (initial 3 months of AID use) to the end of the extension phase.

†Rates of severe hypoglycemia and diabetic ketoacidosis from the United States T1D Exchange were 25.2 and 16.8 per 100 person-years, respectively.[2-3]

‡Hypoglycemia resulting in an adverse event but otherwise not meeting the definition of severe hypoglycemia

§Severe hypoglycemia requiring the assistance of another person due to altered consciousness, and requiring another person to actively administer carbohydrate, glucagon, or other resuscitative actions

‖Hyperglycemia with the presence of polyuria, polydipsia, nausea or vomiting, serum ketones >1.5mmol/L or large/moderate urine ketones, either arterial blood pH <7.30, venous pH <7.24, or serum bicarbonate <15 mmol/L, and treatment provided in a health care facility.

¶Hyperglycemia requiring evaluation, treatment or guidance from intervention site, or hyperglycemia resulting in an adverse event but otherwise not meeting the definition of DKA or prolonged hyperglycemia.

#Meter glucose measuring ≥300mg/dL and ketones >1.0mmol/L. One episode of prolonged hyperglycemia was categorized as a serious adverse event related to the study device (overnight following a carbohydrate treatment for hypoglycemia with intermittent CGM disconnection). Participant was taken to the ER with ketones 3.6mmol/L, was discharged the same day, and fully recovered.

** Other related, but non-glycemic adverse events included infection or irritation at infusion site (5 children). There were 3 other adverse events classified as ketosis that did not meet the DKA or prolonged hyperglycemia definition and were considered device-related. There were 16 events of ketosis that were unrelated to study device or procedure. Other events unrelated

to the study device included viral illnesses (e.g. COVID-19, influenza), bee sting, vomiting, itching, gastrointestinal illness, concussion, febrile seizure, superficial lacerations.

Table S7. Percentage of time (%) in Automated Mode in 3-month intervals during the pivotal phase and extension phase

	**Duration of AID**								
Month	**1-3**	**4-6**	**7-9**	**10-12**	**13-15**	**16-18**	**19-21**	**22-24**	**25-27**
N	80	80	79	79	79	79	45	28	13
**Time in Automated Mode (%)**	96.8 ± 2.6<br>97.8<br>(95.8, 98.5)	95.6 ± 3.8<br>97.0<br>(94.6, 98.0)	96.0 ± 3.5<br>97.5<br>(94.8, 98.6)	95.7 ± 4.9<br>97.7<br>(94.4, 98.7)	95.7 ± 4.8<br>97.2<br>(96.1, 98.0)	96.1 ± 3.9<br>97.6<br>(95.5, 98.5)	95.2 ± 4.4<br>96.9<br>(93.7, 97.9)	92.0 ± 5.5<br>94.2<br>(89.0, 95.8)	94.2 ± 6.9<br>95.9<br>(94.4, 98.3)

Data are shown as mean ± S.D., median (IQR). IQR denotes interquartile range.

Table S8. Insulin requirements and weight changes during the standard therapy, pivotal, and extension phases

	**Standard Therapy Phase**	**Pivotal Study**	**Extension Study**	**Change from Standard Therapy to Extension**	***p*-value**
**Insulin Requirement**					
Total daily insulin (U/kg)	(**n=79**)<br>0.69 ± 0.18,<br>0.69 (0.58, 0.79)	(**n=80**)<br>0.71 ± 0.15,<br>0.70 (0.62, 0.80)	(**n=77**)<br>0.80 ± 0.17,<br>0.76 (0.69, 0.90)	(**n=76**)<br>0.11 ± 0.14,<br>0.08 (0.02, 0.18)	<0.0001[2]
Total daily basal insulin (U/kg)	(**n=80**)<br>0.28 ± 0.12,<br>0.27 (0.20, 0.35)	(**n=80**)<br>0.32 ± 0.10,<br>0.32 (0.25, 0.38)	(**n=77**)<br>0.38 ± 0.11,<br>0.37 (0.31, 0.43)	(**n=77**)<br>0.09 ± 0.12,<br>0.10 (0.03, 0.15)	<0.0001[1]
Total daily bolus insulin (U/kg)	(**n=79**)<br>0.41 ± 0.15,<br>0.39 (0.32, 0.49)	(**n=80**)<br>0.39 ± 0.10,<br>0.38 (0.32, 0.45)	(**n=77**)<br>0.42 ± 0.11,<br>0.40 (0.35, 0.48)	(**n=76**)<br>0.02 ± 0.11,<br>0.00 (-0.05, 0.08)	0.3184[2]
Total daily insulin (U)	(**n=79**)<br>13.7 ± 4.4,<br>13.8 (11.1, 16.1)	(**n=80**)<br>14.1 ± 4.0,<br>14.3 (11.8, 16.4)	(**n=80**)<br>16.4 ± 4.5,<br>16.1 (13.8, 18.2)	(**n=79**)<br>2.7 ± 2.8,<br>2.0 (1.0, 3.8)	<0.0001[2]
Total daily basal insulin (U)	(**n=80**)<br>5.6 ± 2.5,<br>5.3 (3.7, 7.2)	(**n=80**)<br>6.4 ± 2.4,<br>6.5 (4.5, 8.0)	(**n=80**)<br>7.7 ± 2.8,<br>7.5 (6.0, 8.9)	(**n=80**)<br>2.1 ± 2.4,<br>2.3 (0.6, 3.2)	<0.0001[1]
Total daily bolus insulin (U)	(**n=79**)<br>8.1 ± 3.3,<br>8.0 (6.1, 9.9)	(**n=80**)<br>7.7 ± 2.3,<br>7.6 (6.0, 9.0)	(**n=80**)<br>8.7 ± 2.5,<br>8.2 (6.9, 10.1)	(**n=79**)<br>0.6 ± 2.3,<br>0.3 (-0.8, 1.6)	0.0283[2]
Number of boluses (per day)	(**n=79**)<br>6.8 ± 2.8,<br>6.1 (4.9, 8.3)	(**n=80**)<br>7.5 ± 2.7,<br>6.9 (5.5, 8.8)	(**n=80**)<br>7.4 ±2.5,<br>7.3 (5.4, 8.7)	(**n=79**)<br>0.7 ± 2.5,<br>0.5 (-0.8, 1.9)	0.0204[1]
Insulin from user-initiated boluses (%)	(**n=79**)<br>58.7 ± 13.6,<br>59.0 (50.0, 66.9)	(**n=80**)<br>55.0 ± 8.3,<br>55.4 (49.0, 60.1)	(**n=80**)<br>53.5 ± 8.1,<br>53.1 (48.2, 58.9)	(**n=79**)<br>-5.2 ± 13.4,<br>-7.4 (-13.9, 1.0)	0.0001[2]
**Body Weight**					
BMI (kg/m^2)	(**n=80**)<br>16.7 ± 1.5,<br>16.6 (15.5, 17.9)	(**n=77**)<br>16.7 ± 1.4,<br>16.4 (15.7, 17.6)	(**n=62**)<br>16.9 ± 2.3,<br>16.6 (15.8, 17.9)	(**n=62**)<br>0.1 ± 1.8,<br>0.0 (-0.6, 0.7)	0.5347[2]

BMI z-score	**(n=80)**<br>0.74 ± 0.95,<br>0.83 (0.07, 1.40)	**(n=77)**<br>0.76 ± 0.89,<br>0.71 (0.30, 1.34)	**(n=61)**<br>0.04 ± 6.41,<br>0.79 (0.25, 1.27)	**(n=61)**<br>-0.79 ± 6.09,<br>-0.05 (-0.52, 0.28)	0.4032[2]

Data are shown as mean ± S.D., median (IQR). IQR denotes interquartile range.

[1] Unadjusted two-sided p-value for paired t-test.
[2] Two-sided Wilcoxon signed rank test.

Supplementary Figure S1. Subgroup analysis of HbA1c in 3-month intervals stratified by baseline HbA1c.

HbA1c for children in 3-month intervals stratified by baseline HbA1c <8% (left) and ≥8% (right). Dashed line represents the consensus target of HbA1c 7.0%. Error bars show the standard deviation. **p<0.01 and ***p<0.001.

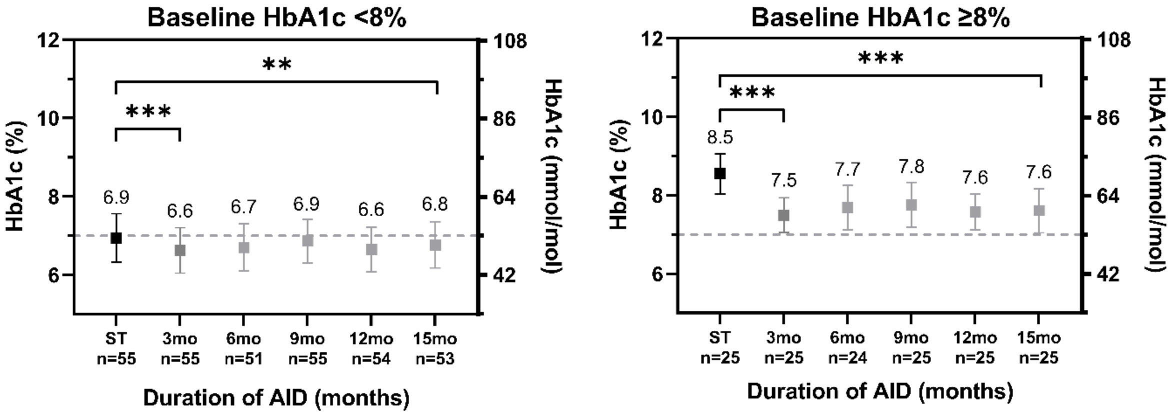

REFERENCES

1. ElSayed NA, Aleppo G, Aroda VR, et al. 6. Glycemic Targets: Standards of Care in Diabetes-2023. *Diabetes Care*. 2023;46(Suppl 1):S97-s110.
2. Foster NC, Beck RW, Miller KM, et al. State of Type 1 Diabetes Management and Outcomes from the T1D Exchange in 2016-2018. *Diabetes technology & therapeutics*. 2019;21(2):66-72.
3. Miller KM, Foster NC, Beck RW, et al. Current state of type 1 diabetes treatment in the U.S.: updated data from the T1D Exchange clinic registry. *Diabetes Care*. 2015;38(6):971-8.
